# Indirect comparison of 48-week efficacy and safety of long-acting cabotegravir and rilpivirine maintenance every 8 weeks with daily oral standard of care antiretroviral therapy in participants with virologically suppressed HIV-1-infection

**DOI:** 10.1186/s12879-022-07243-3

**Published:** 2022-05-04

**Authors:** Vasiliki Chounta, Sonya J. Snedecor, Sterling Wu, Nicolas Van de Velde

**Affiliations:** 1 ViiV Healthcare, Brentford, UK; 2 Pharmerit-an OPEN Health Company, Bethesda, MD USA; 3 GlaxoSmithKline, Collegeville, PA USA

**Keywords:** Anti-HIV agents/administration and dosage, Anti-retroviral agents/therapeutic use, HIV infections/drug therapy, Indirect treatment comparison, Long-acting

## Abstract

**Background:**

Efficacy and safety of long-acting cabotegravir (CAB) + rilpivirine (RPV) every 8 weeks (Q8W) versus daily oral standard of care (SoC) maintenance in treatment-experienced individuals with virologically suppressed human immunodeficiency virus type 1 (HIV-1) has not been directly compared in randomized clinical trials. This analysis aimed to indirectly compare these regimens.

**Methods:**

An adjusted indirect treatment comparison of CAB + RPV Q8W with daily oral SoC was performed, using Phase 3 data from studies of CAB + RPV every 4 weeks (Q4W) vs SoC (ATLAS/FLAIR, n = 591 per group) and a Phase 3b trial of CAB + RPV Q8W vs Q4W (ATLAS-2M [excluding participants with prior CAB + RPV exposure]; n = 327 per group). Eligible participants were virologically suppressed (viral load < 50 HIV-1 ribonucleic acid (RNA) copies/mL), treatment-experienced individuals with HIV-1-infection. Treatment efficacy and safety assessments at Week 48 included virologic suppression and lack of virologic suppression (proportion of participants with plasma HIV-1 RNA < 50 copies/mL or ≥ 50 copies/mL, respectively; both as per FDA snapshot algorithm), CD4-cell count change from baseline, no virologic data, discontinuations due to adverse events (AEs), and overall AEs, serious AEs and Grade 3–5 AEs excluding injection-site reactions. A subgroup analysis stratified by baseline third active drug class was performed.

**Results:**

Baseline characteristics between the Q4W arms of ATLAS/FLAIR and ATLAS-2M showed no significant differences or differences were not judged to be clinically relevant, apart from participants switching from a baseline third active drug class; more participants switched from integrase strand inhibitors in ATLAS/FLAIR, and from non-nucleoside reverse transcriptase inhibitors in ATLAS-2M. Injections of CAB + RPV Q8W showed no significant differences across efficacy and safety outcomes versus daily oral SoC. Univariate subgroup analysis found there were no significant differences on virologic suppression or lack of virologic suppression for any baseline third active drug class subgroup. These results suggest that CAB + RPV Q8W is non-inferior to daily oral SoC.

**Conclusions:**

This analysis supports the therapeutic potential of CAB + RPV Q8W for virologically suppressed people living with HIV-1 infection seeking an alternative maintenance treatment option to daily oral SoC.

*Trial registration:* NCT02938520, NCT02951052, NCT03299049.

**Supplementary Information:**

The online version contains supplementary material available at 10.1186/s12879-022-07243-3.

## Background

The efficacy of human immunodeficiency virus type 1 (HIV-1) treatments has improved steadily over the past few decades, with combination antiretroviral therapy (cART) now being the standard of care (SoC) [1, 2]. Typically, cART is comprised of two nucleoside/nucleotide reverse transcriptase inhibitors (NRTIs) plus a third active drug from a different class [1, 2]. New cART regimens of two (i.e. dolutegravir [DTG] + lamivudine) rather than three drugs have recently been included in guidelines [1].

The primary goal of cART is to reduce HIV-1-associated morbidity and mortality by maximally inhibiting HIV-1 replication, as measured by viral load [1]. An emerging clinical treatment strategy is to switch successfully treated individuals with virologically suppressed HIV-1-infection to maintenance regimens containing fewer drugs, potentially simplifying administration and reducing long-term drug toxicities, among other reasons [3–6]. Currently, all available cART options are administered daily as oral formulations [1]. Although highly efficacious and well tolerated, daily oral ART can affect the well-being of some people living with HIV (PLHIV) due to it being a daily reminder of HIV status and its potential to allow unwanted disclosure of HIV infection [7–10]. In addition, for some PLHIV, maintaining optimal adherence levels can be challenging due to lifestyle factors, forgetfulness, or other reasons [11].

Cabotegravir (CAB), an integrase strand transfer inhibitor (INSTI), has been developed as a long-acting injectable drug used in combination with long-acting injectable rilpivirine (RPV), a non-nucleoside reverse transcriptase inhibitor (NNRTI), for virologically suppressed PLHIV. This combination therapy may improve drug adherence, while reducing the burden and potential emotional impact of daily treatment. The non-inferiority of CAB + RPV LA (long acting) administered every 4 weeks (Q4W) in maintaining virologic suppression compared with daily oral SoC ART has been demonstrated in two pivotal Phase 3 studies (Antiretroviral Therapy as Long-Acting Suppression [ATLAS] and First Long-Acting Injectable Regimen [FLAIR]) as well as in a pooled analysis of these studies [12–14]. Participants receiving CAB + RPV LA reported greater satisfaction with and preference for this regimen over previous oral therapy [12–14]. Additionally, the ATLAS every 2 months (ATLAS-2M) Phase 3b study demonstrated the non-inferiority of CAB + RPV LA (600 mg + 900 mg) administered every 8 weeks (Q8W) versus CAB + RPV (400 mg + 600 mg) Q4W over a 48-week treatment period, showing that virologic suppression can be maintained with a different dose and a reduced frequency of dosing. In ATLAS-2M, study participants preferred the Q8W dosing over daily oral or Q4W dosing [15].

No randomized clinical trial comparing CAB + RPV LA Q8W and daily oral SoC regimens is currently available to allow a direct comparison of efficacy and safety of these maintenance regimens in treatment-experienced individuals with virologically suppressed HIV-1 infection. Therefore, the current analysis aimed to indirectly compare CAB + RPV LA Q8W with daily oral SoC maintenance regimens 48 weeks after treatment switch from SoC.

## Methods

### Study design

An adjusted indirect comparison of CAB + RPV LA Q8W with daily oral SoC was conducted according to the International Society for Pharmacoeconomics and Outcomes Research (ISPOR) guidelines on good research practices for indirect treatment comparisons [16]. To date, the only trial of CAB + RPV LA Q8W is ATLAS-2M which compared this regimen with CAB + RPV LA Q4W; the available trials that compare CAB + RPV LA with daily oral SoC maintenance treatments have used a Q4W CAB + RPV LA regimen (ATLAS and FLAIR). Thus, the evidence base for this analysis of CAB + RPV LA Q8W versus daily oral SoC was necessarily limited to the ATLAS, FLAIR, and ATLAS-2M trials [12, 13, 15]. An anchored Bucher’s frequentist adjusted indirect treatment comparison was used for this analysis of CAB + RPV LA Q8W and oral daily SoC, with CAB + RPV LA Q4W as the common comparator [17]. Since combining studies should only be considered if they are clinically and methodologically similar, we employed the PICOS approach to compare trials in terms of population (P), intervention (I), comparator (C), outcome (O), and study design (S) (Table 1). The ATLAS, FLAIR, and ATLAS-2M trials were all 1:1 randomized, open-label studies, evaluating non-inferiority in efficacy after switching to the intervention of interest in a virologically suppressed population at Week 48 with primary and secondary endpoints being identical between the trials included in the analysis (Table 1).

**Table 1:** Summary of study characteristics of the ATLAS, FLAIR, and ATLAS-2M trials

Trial acronym (Author year)	Study design	Countries/region	Participant eligibility criteria	Intervention/comparator	Endpoints
ATLAS (Swindells 2020) [13]	Randomized, multicenter, parallel-group, open-label, Phase 3	Argentina Australia Canada Europe Mexico Republic of Korea Russian Federation South Africa USA	• On initial or second ARV regimen ≥ 6 months prior to screening • Prior switch only for tolerability/safety, access to medications, or convenience/ simplification, and NOT due to treatment failure • HIV-1 RNA < 50 copies/mL at screening and ≥ 2 HIV-1 RNA measurements < 50 copies/mL in the 12 months prior to screening	• CAB + RPV Q4W • Continued cART SoC therapy	• Primary: % participants with HIV-1 RNA ≥ 50 copies/mL at Week 48 • Key secondary: participants with HIV-1 RNA < 50 copies/mL at Week 48
FLAIR (Orkin 2020) [12]	Randomized, multicenter, open-label, non-inferiority, Phase 3	Canada Europe Japan Russian Federation South Africa USA	• Treatment naïve at screening • 20 weeks induction period with DTG/ABC/3TC • HIV-1 RNA < 50 copies/mL prior to randomization	• CAB + RPV Q4W • DTG + ABC + 3TC	• Primary: % participants with HIV-1 RNA ≥ 50 copies/mL at Week 48 • Key secondary: participants with HIV-1 RNA < 50 copies/mL at Week 48
ATLAS-2M (Overton 2021) [15, 31]	Randomized, multicenter, parallel-group, open-label, non-inferiority, Phase 3	Argentina Australia Canada Europe Mexico North America Republic of Korea Russian Federation South Africa USA	• Uninterrupted SoC regimen ≥ 6 months • 2 documented HIV-1 RNA viral load measurements • < 50 copies/mL in each past 2, 6-month periods and at study entry	• CAB + RPV Q4W^a^ • CAB + RPV Q8W^a^	• Primary: % participants with HIV-1 RNA ≥ 50 copies/mL at Week 48 • Key secondary: participants with HIV-1 RNA < 50 copies/mL at Week 48

*3TC* lamivudine, *ABC* abacavir, *ARV* antiretroviral, *ATLAS* Antiretroviral Therapy as Long-Acting Suppression, *ATLAS-2M* ATLAS every 2 months, *CAB* cabotegravir, *cART* combination antiretroviral therapy, *DTG* dolutegravir, *FLAIR* First Long-Acting Injectable Regimen, *LA* long-acting, *HIV-1* human immunodeficiency virus type 1, *Q4W* every 4 weeks, *Q8W* every 8 weeks, *RNA* ribonucleic acid, *RPV* rilpivirine, *SoC* standard of care

^a^Participants with prior CAB + RPV LA exposure were excluded from the current analysis

### Participants and treatments

Eligible participants from the ATLAS, FLAIR, and ATLAS-2M trials were all treatment-experienced individuals with virologically suppressed HIV-1 infection, and a viral load of < 50 HIV-1 ribonucleic acid (RNA) copies/mL. Participants from FLAIR, ATLAS and ATLAS-2M received oral lead-in therapy with 30 mg CAB + 25 mg RPV once daily for four weeks before long-acting injectable was started to mitigate the risk of any serious side-effects associated with CAB or RPV before administering long-acting injectable regimen. Participants in the ATLAS and ATLAS-2M trials had initiated treatment with any standard daily oral SoC prior to the switch to CAB + RPV LA [12, 13, 15]. Some participants in ATLAS-2M had prior CAB + RPV LA Q4W exposure; these participants were excluded from the current analysis, aiming for similarity in baseline participant characteristics as per ISPOR guidelines.

A pooled analysis of the ATLAS and FLAIR trials was previously conducted [14], and no significant heterogeneity between the two trials in terms of trial or participant characteristics for the treatment effect was found. Pooled data from ATLAS and FLAIR studies (n = 591 per treatment group), and data from participants with no prior CAB + RPV exposure (n = 327 per treatment group) in the ATLAS-2M trial, were used to inform this analysis.

**Fig. 1:**
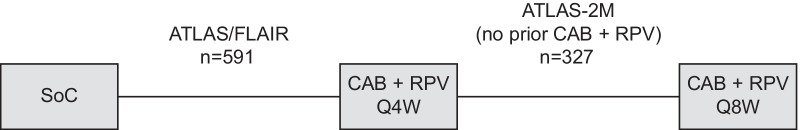
Diagram of studies included in indirect comparison. *CAB* cabotegravir, *Q4W* every 4 weeks, *Q8W* every 8 weeks, *RPV* rilpivirine, *SoC* standard of care, *ATLAS/FLAIR* antiretroviral therapy as long-acting suppression/first long-acting injectable regimen, *ATLAS-2M* antiretroviral therapy as long-acting suppression every 2 months

In addition, aiming to further satisfy the similarity assumption as per ISPOR guidelines, we explored comparability of participants’ demographics and clinical characteristics at study entry between trials. Participants in the FLAIR trial had DTG-based induction therapy prior to the switch to CAB + RPV LA [12]. Pooled data from the ATLAS and FLAIR trials, created overall data for CAB + RPV LA Q4W similar to the CAB + RPV LA Q4W arm of the ATLAS-2M trial (Fig. 1). Due to differences between studies in baseline third agent [12, 13, 15], a subgroup analyses of virologic suppression and lack of virologic suppression stratified by baseline third active drug class were conducted to validate the findings of the main analysis.

This study is a statistical analysis of data from the ATLAS, FLAIR and ATLAS-2M trials and therefore does not require approval from an institutional review board or ethics committee. All trials included in this study were conducted in accordance with the Declaration of Helsinki [18]. All participants provided written informed consent and the protocols were approved by an institutional review board or ethics committee from each study site.

### Study measures

The most frequently reported safety and efficacy outcomes were selected for quantitative comparison as follows: treatment efficacy was assessed for the primary study endpoint, the estimated proportion of participants with HIV-1 RNA ≥ 50 copies/mL according to the FDA snapshot algorithm at Week 48. Other measures of treatment efficacy were assessed as key secondary endpoints: Week 48 virologic suppression (HIV-1 RNA < 50 copies per mL) as per FDA snapshot algorithm, CD4-cell count changes from baseline, and patients with no virologic data at Week 48. Safety was assessed in terms of discontinuations due to adverse events (AEs), and overall AEs, serious AEs, and Grade 3–5 AEs excluding injection-site reactions (ISRs) at Week 48. AEs were analyzed excluding ISRs to compare the events more likely related to systemic exposure to the respective drugs, rather than primarily to the route of administration. In the ATLAS/FLAIR and ATLAS-2M studies, ISRs for both Q4W and Q8W dosing were mostly mild (Grade 1 or 2) and the frequency progressively reduced to Week 48 among study participants, resulting in very few (< 2%) discontinuations, which are included in the discontinuations due to AEs analysis. Comparative efficacy of virologic suppression and lack of virologic suppression were stratified by baseline third active drug class (INSTI, NNRTI, and protease inhibitor [PI]) in a univariate subgroup analysis to assess the relative effects of each treatment class and whether any statistically significant differences that can impact overall outcomes exist.

### Statistical analyses

Assessment of the similarity of population demographic characteristics at baseline and effect of the baseline third active drug class on efficacy, determined by the percentage of < 50 HIV-1 copies/mL in the Q4W arms of the pooled ATLAS/FLAIR trials and ATLAS-2M was carried out using the Wilcoxon Rank Sum Test and the Fisher's Exact Test. The robustness of comparative efficacy conclusions was assessed using alternate summary statistics. Between treatment comparison statistics for ATLAS/FLAIR (Q4W vs SoC) and for ATLAS-2M (Q8W vs Q4W) are shown in Additional file 1: Table S1.

The statistical methodology published by Bucher et al. [17] was then used to calculate relative risk (RR), odds ratio (OR), and risk difference (RD) for the comparison of CAB + RPV LA Q8W versus daily oral SoC, using CAB + RPV Q4W as the common comparator. In this method, a summary statistic of the comparison is expressed on a continuous scale and assumed to have a normal distribution. The mean difference (MD) and RD scales are assumed to be and were normally distributed. The OR and RR scales were transformed to a normally distributed scale using the natural log transformation (lnOR or lnRR). Thus, we have:

$${Indirect \, treatment \, effect} \left[Q8W-SoC\right]=[\text{ln}\,OR/\text{ln}\,RR/RD/MD]\left[Q8W-Q4W\right] ATLAS2M-[\text{ln}\,OR/\text{ln}\,RR/RD/MD]\left[Q4W-SoC\right] ATLAS+FLAIR$$

$${Standard \, errors} \left(SE\right)= \surd \left(SE\_\left(\text{ln}\,OR/\text{ln}\,RR/RD/MD\_ATLAS{2 M}^{2}+SE\left(\text{ln}\,OR/\text{ln}\,RR/RD/MD\right.{)}^{2}\right)\right)$$

## Results

### Baseline demographics and characteristics

A summary of study population demographics and characteristics at baseline for the pooled ATLAS/FLAIR, and ATLAS-2M trials are shown in Table 2. Baseline characteristics between the Q4W arms of the pooled ATLAS/FLAIR and the ATLAS-2M trials showed no significant differences for sex, race, ethnicity, and body mass index; Table 2). A significant difference between treatment groups was found for both median age (ATLAS/FLAIR: 38 years; ATLAS-2M: 41 years; P = 0.0002) and mean CD4 counts (ATLAS/FLAIR: 670.2, ATLAS-2M: 741.0; P = 0.0004); however, as per ISPOR guidelines on assessing similarity for indirect treatment comparisons, although researchers may be able to use statistical information they must rely primarily on clinical judgment of whether differences among studies may affect the comparisons of treatments or make some comparisons inappropriate [16]. An MD in age of 3 years between treatment groups, although statistically significant, is very unlikely to be clinically relevant. Additionally, both ATLAS/FLAIR and ATLAS-2M participants had high CD4-cell count at baseline, both are within the range of a normal CD4-cell count, and therefore the differences are not considered clinically relevant. In addition, it was observed in the individual studies (ATLAS/FLAIR/ATLAS-2M) that efficacy outcomes per age group and CD4-cell count were consistent with the overall population, suggesting these factors were not effect modifiers in the clinical trials used in this comparison. The combined data for the ATLAS/FLAIR trials showed 65% of participants received INSTI treatment and 26% received NNRTIs at baseline; in contrast, data for the ATLAS-2M trial showed 42% of participants received INSTI treatment and 47% received NNRTIs at baseline (Table 2).

**Table 2:** Study population demographic characteristics at baseline for the ATLAS, FLAIR, pooled ATLAS/FLAIR and ATLAS-2M trials

	ATLAS/FLAIR [12–14]		ATLAS-2M [15]		Statistical comparison
	SoC (n = 591)	CAB + RPV Q4W (n = 591)	CAB + RPV Q4W^a^ (n = 327)	CAB + RPV Q8W^a^ (n = 327)	ATLAS/FLAIR vs ATLAS-2M CAB + RPV Q4W
Age (years), median (range)	38 (18, 82)	38 (19, 74)	42 (19, 67)	41 (20, 83)	P = 0.0002^b^
Male, n (%)	423 (72)	429 (73)	252 (77)	254 (78)	P = 0.1563^c^
Race, n (%)					P = 0.0955^d^
White	408 (69)	430 (73)	256 (78)	238 (73)	
Black/African American	133 (23)	109 (18)	45 (14)	57 (17)	
Asian	28 (5)	34 (6)	12 (4)	17 (5)	
Other	20 (3)	18 (3)	14 (4)	15 (5)	
Ethnicity					
Hispanic/Latino, n (%)	74 (13)	63 (11)	42 (13)	54 (17)	P = 0.3309^c^
BMI (kg/m^2^), mean (SD)	25.9 (5.4)	25.7 (4.8)	26.2 (5.2)	26.2 (5.1)	P = 0.1451^b^
CD4-cell count (cells/mm^2^), mean (SD)	670.2 (273.2)	672.7 (264.3)	741.0 (288.8)	688.6 (266.0)	P = 0.0004^b^
CD4-cell count (cells/mm^2^), n (%)					P = 0.0526^d^
< 350	54 (9)	42 (7)	17 (5)	25 (8)	
≥ 350 to < 500	117 (20)	120 (20)	49 (15)	60 (18)	
≥ 500	420 (71)	429 (73)	261 (80)	242 (74)	
Baseline third active class, n (%)					
INSTI	382 (65)	385 (65)	141 (43)	136 (42)	P < 0.0001^d^
NNRTI	155 (26)	155 (26)	156 (48)	151 (46)	
PI	54 (9)	51 (9)	30 (9)	40 (12)	–

All statistical tests compare Q4W groups in ATLAS/FLAIR and ATLAS-2M

*ATLAS* Antiretroviral Therapy as Long-Acting Suppression, *ATLAS-2M* ATLAS every 2 months, *BMI* body mass index, *CAB* cabotegravir, *cART* combination antiretroviral therapy, *FLAIR* First Long-Acting Injectable Regimen, *HIV-1* human immunodeficiency virus type 1, *INSTI* integrase strand inhibitor, *IQR* interquartile range, *m* months, *NA* not applicable, *NNRTI* non-nucleoside reverse transcriptase inhibitor, *PI* protease inhibitor, *Q4W* every 4 weeks, *Q8W* every 8 weeks, *RNA* ribonucleic acid, *RPV* rilpivirine, *SD* standard deviation, *SoC* standard of care

^a^Participants with prior CAB + RPV LA exposure were excluded

^b^Wilcoxon Rank Sum test

^c^Fisher's exact test

^d^Chi-square test

### Outcomes data from the pooled trials

A summary of 48-week efficacy and safety data from the pooled ATLAS/FLAIR and ATLAS-2M trials used for the indirect treatment comparison is shown in Table 3. Over 90% of participants in all four arms of the pooled ATLAS/FLAIR and ATLAS-2M trials achieved < 50 copies/mL of HIV-1 RNA at 48 weeks. All four treatment groups in ATLAS/FLAIR and ATLAS-2M reported a 2% rate of HIV-1 RNA ≥ 50 copies/mL. The percentage of patients with no virologic data at Week 48 was < 7% across all four treatment groups. Discontinuations due to AEs, including ISRs occurred in fewer than 5% of participants across all trials and treatment arms. Changes from baseline at Week 48, in mean CD4-cell count were small across all trials and treatment arms (− 19.2 to 48.2 cells/μL). Overall AEs excluding ISRs occurred in a similar percentage of participants receiving CAB + RPV Q4W in the pooled ATLAS/FLAIR and ATLAS-2M trials (86.3% vs 86.2%, respectively). Likewise, the percentages of participants receiving SoC and CAB + RPV Q8W who experienced AEs excluding ISRs were similar (75.3% vs 77.7%, respectively). Serious AEs excluding ISRs occurred in approximately 5% or fewer and Grade 3–5 AEs occurred in 8% or fewer of participants across all trials and arms (Table 3).

**Table 3:** Summary of 48-week efficacy and safety data from the pooled ATLAS/FLAIR and ATLAS-2M trials considered for the indirect treatment comparison (full analysis set)

	ATLAS/FLAIR [12–14]		ATLAS-2M [15]	
	SOC (n = 591)	CAB + RPV Q4W (n = 591)	CAB + RPV Q4W^a^ (n = 327)	CAB + RPV Q8W^a^ (n = 327)
HIV-1 RNA < 50 copies/mL, n (%)^b^	558 (94.4)	550 (93.1)	300 (91.7)	306 (93.6)
HIV-1 RNA ≥ 50 copies/mL, n (%)^b^	10 (1.7)	11 (1.9)	5 (1.5)	5 (1.5)
CD4-cell count (cells/μl), mean (SD) change from baseline	48.2 (182.1)	24.5 (191.3)	− 19.2 (204.9)	− 0.7 (150.6)
No virologic data at week 48^c^, n (%)^b^	23 (3.9)	30 (5.1)	22 (6.7)	16 (4.9)
Discontinuation due to AEs, n (%)^d^	7 (1.2)	19 (3.2)	11 (3.4)	6 (1.8)
Any AE (excluding ISR), n (%)	445 (75.3)	510 (86.3)	282 (86.2)	254 (77.7)
Serious AEs (excluding ISR), n (%)	26 (4.4)	31 (5.2)	11 (3.4)	16 (4.9)
Grade 3–5 AEs (excluding ISR) maintenance phase	35 (5.9)	47 (8.0)	20 (6.1)	16 (4.9)

*AE* adverse event, *ATLAS* Antiretroviral Therapy as Long-Acting Suppression, *ATLAS-2M* ATLAS every 2 months, *CAB* cabotegravir, *FLAIR* First Long-Acting Injectable Regimen, *HIV-1* human immunodeficiency virus type 1, *ISR* injection-site reaction, *Q4W* every 4 weeks, *Q8W* every 8 weeks, *RNA* ribonucleic acid, *RPV* rilpivirine, *SD* standard deviation, *SoC* standard of care, *LA* Long-acting

^a^Participants with prior CAB + RPV LA exposure were excluded

^b^As per the FDA snapshot algorithm

^c^These include discontinuations due to AEs and other reasons such as: lost to follow-up, protocol deviations, investigator decision, lack of efficacy etc.

^d^These include participants with no virologic data at Week 48 who discontinued due to AEs

### Results from the indirect comparison

**Fig. 2:**
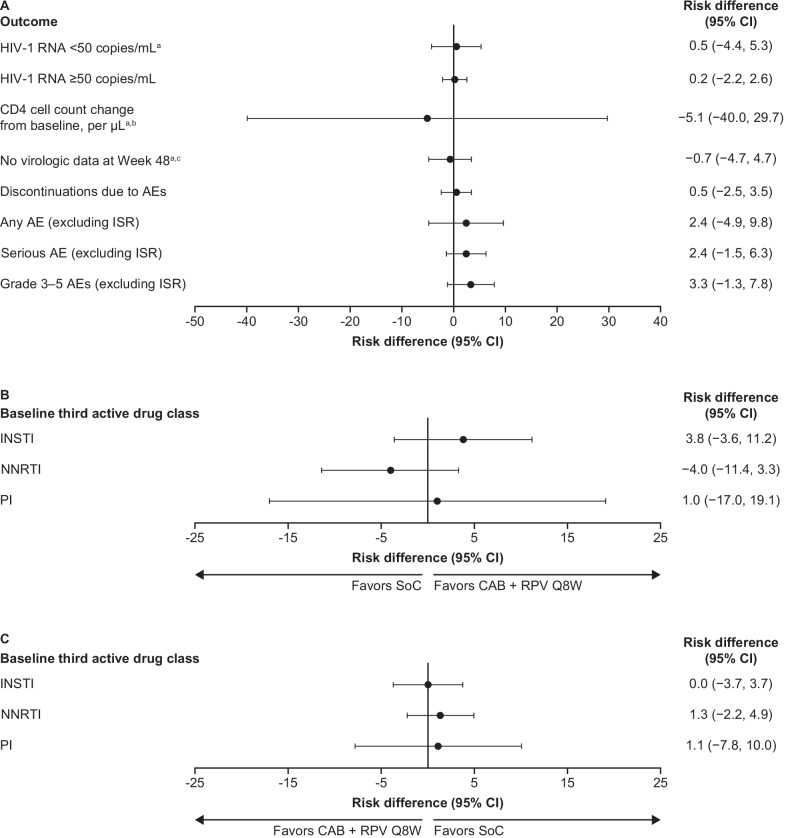
Summary of risk difference results of the indirect comparison of CAB + RPV LA Q8W relative to SoC **A** by outcome, **B** virologic suppression (HIV RNA < 50 copies/mL as per FDA snapshot algorithm) results stratified by baseline third active drug class, and **C** lack of virologic suppression (HIV ≥ 50 copies/mL as per FDA Snapshot Algorithm) results stratified by baseline third active drug class. ^a^Favors CAB + RPV Q8W. ^b^Mean difference. ^c^Values could not be calculated for RR and OR as value for SoC in ATLAS/FLAIR was 0. *AE* adverse event, *ATLAS/FLAIR* antiretroviral therapy as long-acting suppression/first long-acting injectable regimen, *CAB* cabotegravir, *CI* confidence interval, *HIV-1* human immunodeficiency virus type 1, *INSTI* integrase strand inhibitor, *ISR* injection-site reaction, *LA* Long-acting, *NNRTI* non-nucleoside reverse transcriptase inhibitor, *OR* Odds ratio, *PI* protease inhibitor, *Q8W* every 8 weeks, *RNA* ribonucleic acid, *RPV* rilpivirine, *RR* Relative risk, *SoC* standard of care

Results of the indirect comparison of CAB + RPV LA Q8W with daily oral SoC are summarized in Fig. 2A and Table 4. The efficacy outcomes analyzed were virologic suppression (HIV-1 RNA < 50 copies/mL), HIV-1 RNA ≥ 50 copies/mL, no virologic data at Week 48, and change from baseline in CD4-cell count. There were no statistically significant differences in any of the key efficacy outcomes analyzed for CAB + RPV LA Q8W compared with daily oral SoC at Week 48. Similarly, there were no statistically significant differences in any safety outcome analyzed, discontinuations due to AEs, and any AEs, serious AEs, and Grade 3–5 AEs not related to ISRs, for CAB + RPV LA Q8W compared with daily oral SoC at Week 48. This was consistent across all comparative effect measures assessed, including RR, OR, and RD, and MD for CD4-cell count change from baseline (Table 4).

**Table 4:** Summary of results of the indirect comparison of CAB + RPV LA Q8W relative to SoC

	Comparative effect measure (95% CI)		
	Relative risk	Risk difference, %	Odds ratio
HIV-1 RNA < 50 copies/mL at Week 48	1.01 (0.95, 1.06)	0.5 (− 4.40, 5.3)	1.04 (0.49, 2.22)
HIV-1 RNA ≥ 50 copies/mL at Week 48	1.10 (0.25, 4.90)	0.2 (− 2.20, 2.60)	1.10 (0.24, 5.03)
CD4 cell count change from baseline, per μL^a^ at Week 48	–	− 5.1 (− 40.0, 29.7)	–
No virologic data at Week 48	0.95 (0.42, 2.15)	− 0.7 (− 4.90, 3.60)	0.94 (0.40, 2.24)
Discontinuations due to AEs at Week 48	1.48 (0.40, 5.46)	0.5 (− 2.5, 3.5)	1.49 (0.39, 5.65)
Any AE (excluding ISR) maintenance phase	1.03 (0.94, 1.13)	2.4 (− 4.90, 9.80)	1.15 (0.69, 1.90)
Serious AE (excluding ISR) maintenance phase	1.73 (0.73, 4.11)	2.4 (− 1.50, 6.3)	1.78 (0.72, 4.40)
Grade 3–5 AEs (excluding ISR) maintenance phase	1.68 (0.78, 3.61)	3.3 (− 1.3, 7.8)	1.74 (0.77, 3.92)
HIV-1 RNA < 50 copies/mL at Week 48 by baseline third active drug class			
INSTI	1.04 (0.96, 1.13)	3.8 (− 3.6, 11.2)	1.62 (0.57, 4.60)
NNRTI	0.96 (0.89, 1.04)	− 4.0 (− 11.4, 3.3)	0.50 (0.13, 1.99)
PI	1.01 (0.83, 1.24)	1.0 (− 17.0, 19.1)	0.96 (0.11, 8.12)
HIV-1 RNA ≥ 50 copies/mL at Week 48 by baseline third active drug class			
INSTI	1.03 (0.13, 7.97)	0 (− 3.7, 3.7)	1.03 (0.13, 8.27)
NNRTI	2.07 (0.08, 52.49)	1.3 (− 2.2, 4.9)	2.09 (0.08, 54.86)
PI^b^	–	1.1 (− 7.8, 10.0)	–

*AE* adverse event, *ATLAS/FLAIR* antiretroviral therapy as long-acting suppression/first long-acting injectable regimen, *CAB* cabotegravir, *CI* confidence interval, *HIV-1* human immunodeficiency virus type 1, *INSTI* integrase strand inhibitor, *ISR* injection-site reaction, *LA* long-acting, *NNRTI* non-nucleoside reverse transcriptase inhibitor, *OR* odds ratio, *PI* protease inhibitor, *Q8W* every 8 weeks, *RNA* ribonucleic acid, *RPV* rilpivirine, *RR* relative risk, *SoC* standard of care

^a^Mean difference

^b^Values could not be calculated for RR and OR as value for SoC in ATLAS/FLAIR was 0

### Univariate subgroup analysis

The CAB + RPV LA Q8W versus daily oral SoC virologic suppression comparisons were stratified by baseline third active drug class to understand the potential impact on the study conclusions of the unmatched distribution of participants switching from INSTIs and NNRTIs to study treatment between the pooled ATLAS/FLAIR and ATLAS-2M trials (65% vs 42% of participants received INSTI, and 26% vs and 47% received NNRTIs in the trials, respectively). Comparative effect measures consistently showed no statistically significant differences in virologic suppression or lack of virologic suppression for any of the baseline third active drug classes assessed (Table 4; Fig. 2B and C). This suggests that baseline third active drug class does not impact the findings of the main analysis.

## Discussion

Previous research has established the non-inferiority of CAB + RPV LA Q4W in the maintenance of virologic suppression compared with daily oral SoC (ATLAS and FLAIR) [12, 13], as well as the non-inferiority of CAB + RPV LA Q8W compared with Q4W dosing (ATLAS-2M) [15]. To date, however, there have been no randomized clinical trials comparing the efficacy and safety of CAB + RPV LA Q8W with daily oral SoC ART in virologically suppressed PLHIV on maintenance treatment regimens. The current analysis indirectly compared 48-week treatment outcomes between CAB + RPV LA Q8W with daily oral SoC ART, showing that there are no statistically significant differences in key efficacy or safety outcomes, across all comparative effect measures assessed. The results of the current analysis, therefore, suggest that CAB + RPV LA Q8W is comparable to daily oral SoC in terms of expected efficacy and key safety outcomes.

While indirect comparisons provide useful insights, they cannot replace evidence from head-to-head studies, which remain the gold standard. In the absence of a head-to-head study, this indirect comparison can be used to compare treatment outcomes across studies, supporting decisions about best choices for ART treatment [19]. A head-to-head study assessing non-inferiority in efficacy of CAB + RPV Q8W versus bictegravir/emtricitabine/tenofovir alafenamide is currently planned and if successful will validate the findings of this analysis. It is important to appreciate the limitations of indirect comparisons, such as how clinically and methodologically similar the trials being compared are. This can be assessed statistically, but also requires clinical judgment to determine whether differences among studies may affect the clinical interpretation of such comparisons [16]. In the current analysis, there was an unmatched distribution of participants switching from INSTIs and NNRTIs to study treatment between the pooled ATLAS/FLAIR and ATLAS-2M trials, which had the potential to be clinically relevant. Therefore, a subgroup analysis on virologic suppression was carried out to assess any potential effects of this unmatched distribution. The subgroup analysis found that there were no statistically significant differences on virologic suppression or lack of virologic suppression for any of the baseline third active drug classes assessed, implying that the overall conclusions of the main analyses are robust and unaffected by differences in baseline third active drug class across trials. The findings of the indirect comparisons made in this study should nonetheless be interpreted with caution due to the general limitations associated with this type of comparison.

The univariate analysis subgroup analysis showed that differences between studies in baseline third agent did not have any clinically meaningful impact on the main analysis findings. As other baseline demographics and characteristics did not appear to be significantly different between trials or treatment arms, including other clinical factors in a multivariate analysis would be unlikely to change the results of the main analysis; therefore, conducting such an analysis was considered beyond the scope of the current indirect treatment comparison.

More convenient regimens with less frequent dosing requirements will help address some of the remaining challenges related to antiretroviral treatment, including suboptimal adherence, HIV-related stigma, and the need for daily dosing [15, 20–23]. Adherence to ART for patients with HIV-1 is essential to achieve durable clinical outcomes and minimize drug resistance and lack of virologic suppression [4]. Even with one pill a day, adherence is less than optimal, over one quarter of PLHIV on ART experience episodes of non-adherence [24–26]. Surveys conducted in the United States have shown that for populations including people who inject drugs and PLHIV with adherence issues there is great interest in switching to treatments with less frequent dosing [24, 27, 28]. A recent international survey found that although general satisfaction with daily ART was high, there were still a number of emotional challenges associated with it, including the stress and pressure of taking medication at the right time every day, concealment of HIV medication, daily treatment being a constant reminder of HIV status, and treatment disrupting and limiting daily life [29]. Furthermore, the long-acting attribute was ranked among the most valued treatment attributes, second only to long-term safety concerns and adverse events [30]. The results of the present analysis suggest that CAB + RPV LA dosed every 8 weeks is non-inferior to current SoC oral ART in maintaining HIV viral suppression. Given the preference of participants in the ATLAS-2M study for the Q8W dosing regimen, this treatment regimen appears to offer a promising future option for long-term successful ART [15].

## Conclusion

Long-acting injectable CAB + RPV dosed Q8W showed no statistically significant differences in terms of efficacy and safety compared with current HIV Treatment Guideline-recommended daily oral cART regimens in treatment-experienced, virologically suppressed PLHIV. This analysis supports the therapeutic potential of CAB + RPV LA Q8W for PLHIV who seek an alternative maintenance treatment option to daily oral SoC.

## Supplementary Information

**Additional file 1: Table S1.** Statistics for between treatment comparison (treatment 1 vs. treatment 2).

## Data Availability

The majority of the data included in this study are previously published or included in this manuscript. For unpublished clinical data used in this study, information on ViiV Healthcare’s data sharing commitments and how to request access to ViiV Healthcare sponsored studies can be found at www.clinicalstudydatarequest.com.

## Abbreviations

AE: Adverse events
ARV: Antiretroviral
ATLAS: Antiretroviral Therapy as Long-Acting Suppression
ATLAS-2M: Antiretroviral Therapy as Long-Acting Suppression every 2 months
CAB: Cabotegravir
cART: Combination antiretroviral therapy
CI: Confidence interval
DTG: Dolutegravir
FLAIR: First Long-Acting Injectable Regimen
HIV-1: Human immunodeficiency virus type 1
INSTI: Integrase strand inhibitor
ISPOR: International Society for Pharmacoeconomics and Outcomes Research
ISR: Injection-site reaction
LA: Long-acting
MD: Mean difference
NRTI: Nucleoside/nucleotide reverse transcriptase inhibitor
NNRTI: Non-nucleoside reverse transcriptase inhibitor
OR: Odds ratio
PLHIV: People living with HIV
PI: Protease inhibitor
Q4W: Every 4 weeks
Q8W: Every 8 weeks
RD: Risk difference
RNA: Ribonucleic acid
RPV: Rilpivirine
RR: Relative risk
SoC: Standard of care
