# Supplementary material for: Indirect comparison of 48-week efficacy and safety of long-acting cabotegravir and rilpivirine maintenance every 8 weeks with daily oral standard of care antiretroviral therapy in participants with virologically suppressed HIV-1-infection
Source: BMC Infect Dis. 2022 May 4;22:428. doi: 10.1186/s12879-022-07243-3 (PMC9066757; doi:10.1186/s12879-022-07243-3)
Supplement: Supplementary file 1 — Additional file 1: Table S1. Statistics for between treatment comparison (treatment 1 vs. treatment 2). [file 12879_2022_7243_MOESM1_ESM.docx]

**Indirect comparison of 48-week efficacy and safety of long-acting cabotegravir and rilpivirine maintenance every 8 weeks with standard of care in virologically suppressed HIV-1-infected participants**

Vasiliki Chounta,^1^ Sonya J Snedecor^2^, Sterling Wu^3^, Nicholas Van de Velde^1^

^1^ViiV Healthcare, Brentford, UK; ^2^Pharmerit International, Bethesda, MD, USA; ^3^GlaxoSmithKline, Collegeville, PA, USA.

**Table S1.** Statistics for between treatment comparison (treatment 1 vs. treatment 2)

| **Binary outcomes** | **Mean statistic** | **Standard error** |
| --- | --- | --- |
| **Odds ratio (OR)** | $\frac{r_{2}/\left( n_{2}-r_{2} \right)}{r_{1}/\left( n_{1}-r_{1} \right)}$ | $\sqrt{\frac{1}{r_{1}}+\frac{1}{\left( n_{1}-r_{1} \right)}+\frac{1}{r_{2}}+\frac{1}{\left( n_{2}-r_{2} \right)}}$ |
| **Risk ratio or  relative risk (RR)** | $\frac{r_{2}/n_{2}}{r_{1}/n_{1}}$ | $\sqrt{\frac{1}{r_{1}}-\frac{1}{n_{1}}+\frac{1}{r_{2}}-\frac{1}{n_{2}}}$ |
| **Risk difference (RD)** | $\frac{r_{2}}{n_{2}}-\frac{r_{1}}{n_{1}}$ | $\sqrt{\frac{\frac{r_{1}}{n_{1}}\left( 1-\frac{r_{1}}{n_{1}} \right)}{n_{1}}+\frac{\frac{r_{2}}{n_{2}}\left( 1-\frac{r_{2}}{n_{2}} \right)}{n_{2}}}$ |
| **Mean difference (MD)** | $\left( {endpoint mean}_{2}-{baseline mean}_{2} \right)-\left( {endpoint mean}_{1}-{baseline mean}_{1} \right)$ | $\sqrt{{SE}_{diff tx 1}^{2}+{SE}_{diff tx 2}^{2}}$ |

r = number of people with event; n = total number of people; tx = treatment
